# Inhibition of TREM-2 Markedly Suppresses Joint Inflammation and Damage in Experimental Arthritis

**DOI:** 10.3390/ijms23168857

**Published:** 2022-08-09

**Authors:** Alexander B. Sigalov

**Affiliations:** SignaBlok, Inc., P.O. Box 4064, Shrewsbury, MA 01545, USA; sigalov@signablok.com; Tel.: +1-203-505-3807

**Keywords:** triggering receptors expressed on myeloid cells, TREM-1, TREM-2, inflammation, innate immunity, signal transduction, macrophages, cytokines, nanomedicine, drug delivery systems, rheumatoid arthritis

## Abstract

The triggering receptors expressed on myeloid cells (TREMs) are a family of activating immune receptors that regulate the inflammatory response. TREM-1, which is expressed on monocytes and/or macrophages and neutrophils, functions as an inflammation amplifier and plays a role in the pathogenesis of rheumatoid arthritis (RA). Unlike TREM-1, the role in RA of TREM-2, which is expressed on macrophages, immature monocyte-derived dendritic cells, osteoclasts, and microglia, remains unclear and controversial. TREM-2 ligands are still unknown, adding further uncertainty to our understanding of TREM-2 function. Previously, we demonstrated that TREM-1 blockade, using a ligand-independent TREM-1 inhibitory peptide sequence GF9 rationally designed by our signaling chain homooligomerization (SCHOOL) model of cell signaling, ameliorates collagen-induced arthritis (CIA) severity in mice. Here, we designed a TREM-2 inhibitory peptide sequence IA9 and tested it in the therapeutic CIA model, either as a free 9-mer peptide IA9, or as a part of a 31-mer peptide IA31 incorporated into lipopeptide complexes (IA31-LPC), for targeted delivery. We demonstrated that administration of IA9, but not a control peptide, after induction of arthritis diminished release of proinflammatory cytokines and dramatically suppressed joint inflammation and damage, suggesting that targeting TREM-2 may be a promising approach for the treatment of RA.

## 1. Introduction

Rheumatoid arthritis (RA) is an autoimmune and inflammatory disease that affects 0.24–1% of the world population [1,2]. Uncontrolled joint inflammation in RA results in cartilage damage and bone destruction and, eventually, in disability [3]. Life expectancy of patients with RA is reduced by 3–18 years [4] and 80% of RA patients are disabled after 20 years [5]. Despite recent advances [1,6,7], there is no cure for RA yet and 30–50% of RA patients do not respond, or respond poorly, to the first-line standard treatments [8,9], and, among those who respond, 50% relapse shortly after treatment cessation [9], showing an urgent need for new therapies.

Myeloid cells, including macrophages, play a central role in the pathogenesis of RA [10,11]. The abundance and activation of macrophages in the inflamed synovial membrane have been demonstrated to significantly correlate with the severity of RA [10,12,13]. Only those therapies that reduce the number of synovial sublining macrophages are likely to be clinically meaningful [14]. Proinflammatory cytokines, such as tumor necrosis factor-α (TNFα), interleukin-1 (IL-1), and IL-6, as well as macrophage colony-stimulating factor (M-CSF, also known as CSF-1) are involved in macrophage development, activation, growth, and differentiation, representing promising targets in RA [15]. Blocking IL-6 is known to decrease the number of osteoclasts [16] and inhibit joint damage in collagen-induced arthritis (CIA) [17], as well as in human RA [18]. Several biologics that block specific cytokines were approved to treat RA, including blockers of TNFα (e.g., Humira^®^ and Remicade^®^) and IL-1 receptor (Kineret^®^). Due to excessive immunosuppression, the use of these and similar agents can cause fatal infections, malignancies and septic arthritis [19,20,21]. In RA patients with a high baseline TNF, a higher dose of Remicade^®^ is necessary, whereas lower doses are sufficient for those with a low baseline TNF [22,23], leading to the critical need for personalization [24]. This further outlines an immediate need for efficient, safe and well-tolerable RA therapy. Importantly, targeted delivery, such as therapy to myeloid cells of interest (e.g., macrophages) would not only strike the cells that mediate or amplify most of the permanent tissue destruction but also spare other cells that do not affect joint damage [13,25].

Triggering receptors expressed by myeloid cells 1 and 2 (TREM-1 and TREM-2, respectively) are involved in inflammation and activate myeloid cells through their signaling partner, DAP-12 [26]. TREM-1, which is expressed on monocytes, macrophages and neutrophils, mediates release of TNFα, IL-1β, IL-6, and CSF-1 [27,28], and is highly upregulated in the synovium of RA patients [27]. In experimental arthritis, therapeutic inhibition of TREM-1 ameliorates disease [29,30] and, importantly, can blunt excessive inflammation without affecting pathogen clearance [31]. Certain macrophages and neutrophils express both TREM-1 and TREM-2, whereas dendritic cells, osteoclasts, and microglia exhibit predominant expression of TREM-2 [26]. While the detrimental role of TREM-1 in inflammatory diseases, including RA, has been well established in most studies [32], that of TREM-2 has been largely controversial. Contrarily to TREM-1, that acts as an inflammation amplifier, TREM-2 has been shown to act either as a negative [33,34,35,36,37,38] or positive [39,40,41,42] regulator of inflammation in various inflammatory diseases.

High upregulation of TREM-2 in active RA synovium and its subsequent downregulation in inactive RA suggest a role of TREM-2 in RA-induced inflammation [43]. TREM-2 has been shown to be upregulated in the synovial tissue of rats with CIA [44]. However, to the author’s knowledge, no studies have yet investigated the effect of inhibition of TREM-2 in experimental arthritis.

Similarly to TREM-1, numerous molecules were proposed as potential ligand(s) for TREM-2, ranging from various anionic molecules, such as phospholipids and proteoglycans, to apolipoproteins (apos) and heat shock proteins [45]. This suggests that the actual nature of the TREM-2 ligand(s) and mechanisms of TREM-2 signaling are still not yet well understood, impeding the development of clinically relevant inhibitors of TREM-2.

**Figure 1:**
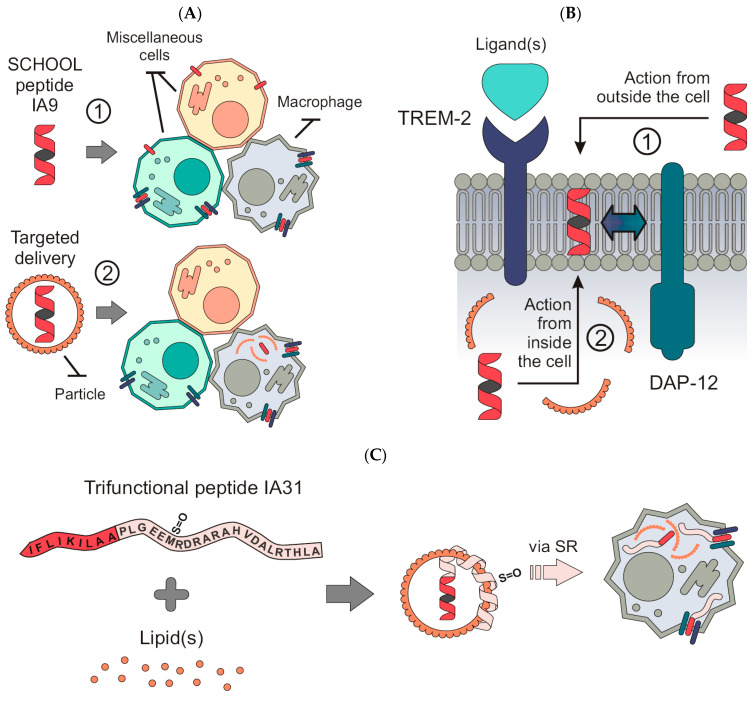
Schematic depiction for the proposed concepts of ligand-independent TREM-2 inhibition and macrophage-targeted drug delivery. (**A**) Peptide IA9 self-inserts into the cell membrane from outside and inhibits TREM-2 on any TREM-2-expressing cell (1). IA9 delivered into macrophages self-inserts into the cell membrane from inside and inhibits TREM-2 expressed on these cells (2). (**B**) Similar to SCHOOL peptide inhibitors of other cell receptors [48], IA9 employs ligand-independent mechanisms of action and blocks interactions between TREM-2 and its signaling partner DAP-12 in the cell membrane. (**C**) Schematic representation of a trifunctional peptide IA31 capable of formation of lipopeptide complexes (IA31-LPC) upon interaction with lipids. Due to sulfoxidized methionine in the PA22 domain, IA31-LPC deliver IA31 to cells (e.g., macrophages) via interaction with scavenger receptor (e.g., type A scavenger receptor, SR-A), where the released IA31 inhibits TREM-2.

In the present study, we used the basic molecular principles of the signaling chain homooligomerization (SCHOOL) model of multichain immune recognition receptor (MIRR)-mediated cell signaling [46,47,48] to rationally design the peptide sequence IA9 for inhibition of TREM-2, a member of the MIRR family (Figure 1A). This nonapeptide employs a novel, ligand-independent mechanism of inhibition and can reach its site of action in the cell membrane from both outside and inside the cell (Figure 1B). As for other ligand-independent peptide inhibitors of various cell receptors (SCHOOL peptides) [48], this mechanism of action not only overcomes the uncertainty of TREM-2 ligand(s), but also allows the use of IA9, either in the form of free peptide, to target TREM-2 on virtually all TREM-2-expressing cells (Figure 1A, Route 1), or formulated into self-assembling lipopeptide complexes (LPC) that mimic human high density lipoproteins (HDL) for targeted intracellular delivery of IA9 to macrophages (Figure 1A, Route 2). To formulate IA9 sequence-containing LPC, we applied a strategy similar to that previously used to design targeted TREM-1 peptide therapy for arthritis [30] and combined the TREM-2 inhibitory peptide sequence IA9 with the 22 amino acid residues long peptide sequence of the apo A-I helix 6 (PA22) (Figure 1C). The resulting peptide IA31 interacts with lipids forming nanosized LPC (IA31-LPC). Due to a sulfoxidized methionine residue in the PA22 domain, these LPC provide targeted delivery of IA31 to cells (e.g., macrophages) via interaction with scavenger receptors (e.g., type A scavenger receptor, SR-A, most abundantly expressed on macrophages [49]) (Figure 1C). Interestingly, SR-A plays a role in excessive synovial osteoclastogenesis, a hallmark of RA [50]. SR-A is mainly involved in an early phase of this process and its level of expression declines during osteoclast differentiation [51]. 

We demonstrated, for the first time, that in the CIA mouse model, IA9 and IA31-LPC systemically administered after induction of arthritis both markedly suppress the rate of disease progression and joint inflammation, diminish release of plasma and joint proinflammatory cytokines and CSF-1, and significantly decrease synovial tissue sublining CD68, F4/80, TREM-1 and TREM-2 expression. We also comparatively studied TREM-1 inhibitory SCHOOL peptide sequence GF9 in the form of free peptide GF9, and formulated into macrophage-targeted LPC, as part of a trifunctional peptide GA31 (GA31-LPC) [30]. We showed that IA9 and IA31-LPC tend to have higher efficacy in the therapeutic CIA model used in this study compared to that of GF9 and GA31-LPC. Collectively, our data suggest that TREM-2 inhibition, using ligand-independent inhibitory SCHOOL peptides, can be a safe, effective and well-tolerable alternative therapy for the treatment of RA.

## 2. Results

### 2.1. Reduction of Inflammation and Suppression of Arthritis in a Therapeutic CIA Model 

Previously [30], we used our proposed molecular mechanisms of transmembrane signaling [46,47] and targeted drug and imaging agent delivery [52,53] to design TREM-1 inhibitory peptide sequence GF9, and demonstrated that prophylactic administration of GF9 as a free peptide GF9 or delivered in the HDL-mimicking macrophage-targeted LPC formulations, efficiently reduces arthritis progression and protects against bone and cartilage damage in mice with CIA, the most widely studied autoimmune model of RA [54]. 

In this study, we used the same concepts to design free and LPC-bound TREM-2 inhibitory IA9 sequences (Figure 1), evaluated their anti-inflammatory and anti-arthritic effects in the therapeutic CIA mouse model and compared them with those of free and LPC-bound GF9 sequence-based inhibitors of TREM-1. 

**Figure 2:**
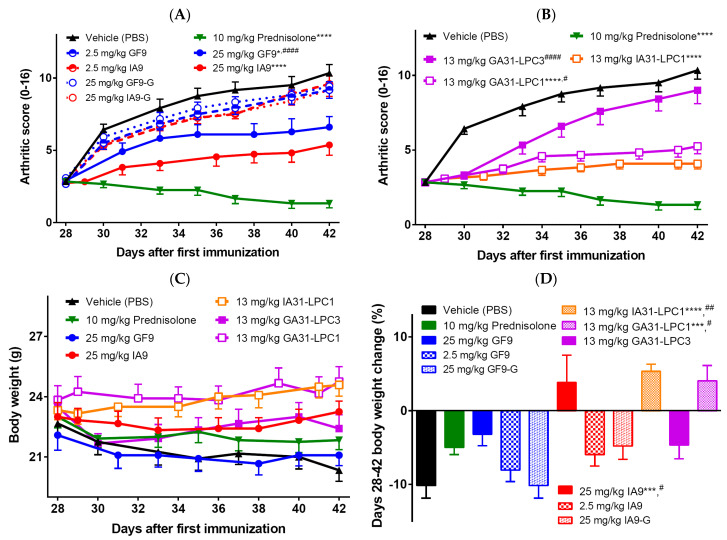
Effect of therapeutic treatment with free (**A**) and LPC-bound (**B**) IA9 and GF9 sequences on the severity of collagen-induced arthritis (**A**,**B**) and body weight (**C**,**D**). Data are shown as mean ± SEM (*n* = 12 mice per group). *, *p* < 0.05; ***, *p* < 0.001; and ****, *p* < 0.0001 versus vehicle. ^#^, *p* < 0.05; ^##^, *p* < 0.01; and ^####^, *p* < 0.0001 versus prednisolone.

When systemically (intraperitoneally, i.p.) administered at 25 mg/kg, IA9, but not a control peptide IA9-G, ameliorated arthritis severity compared to vehicle-treated mice (Figure 2A). The difference between the IA9- and vehicle-treated groups started on day 29 and continued until day 42 (Figure 2A). Therapeutic treatment with IA9, but not vehicle or IA9-G, significantly reduced the arthritic score by day 42 showing the anti-arthritic effect not significantly different from that of 10 mg/kg oral prednisolone used as a positive control (Figure 2A). No therapeutic activity was observed for 2.5 mg/kg IA9 suggesting its effect is dose dependent. Therapeutic effect has also been observed for GF9 at a dose of 25 but not 2.5 mg/kg administered daily, starting day 28 for 14 consecutive days (Figure 2A). In contrast to the previously demonstrated prophylactic effect of 25 mg/kg of GF9 in CIA that was not significantly different from that of 0.1 mg/kg dexamethasone used as positive control [30], the therapeutic effect of 25 mg/kg GF9 observed here was significantly lower compared to that of the positive control (Figure 2A).

To test whether incorporation of TREM-2 inhibitory peptide sequence IA9 as a part of trifunctional peptide IA31 (Figure 1C) into macrophage-targeted LPC (IA31-LPC) affects its therapeutic efficacy in CIA, we used IA31 and POPC to prepare IA31-LPC1 with a mean particle diameter of 94 nm and polydispersity index (PDI) < 0.2 indicating monodispersity of these particles. GA31-LPC1 particles of similar size and size distribution (a mean particle diameter of 96 nm and PDI < 0.2) were also prepared and studied comparatively. 

Earlier studies of liposomes loaded with superoxide dismutase in arthritic rats [55,56] demonstrated that small-sized liposomal formulations have significant advantages over large-sized formulations in terms of localization at arthritic sites and anti-arthritic activity. Recent studies of dexamethasone-loaded liposomes showed that the smallest liposomes (75 nm mean diameter with PDI < 0.2) were the ones that resulted in the higher anti-arthritic efficacy in arthritic rats in terms of suppression of paw thickness, and reduction of arthritic scores, proinflammatory cytokines and transaminase levels compared to two other liposomal formulations with similar dexamethasone content but larger sizes (150 and 300 nm mean diameters; both with PDI < 0.2) [57]. To test a possible particle size effect on the therapeutic activity of the LPC-based formulations used in this study, GA31-LPC3 with a mean particle diameter of 140 nm and PDI < 0.2 were prepared using GA31, POPC, cholesterol, and cholesteryl oleate, and studied comparatively. 

IA31-LPC1, GA31-LPC1 and GA31-LPC3 were i.p. administered (all at the same dose of 13 mg peptide/kg) daily starting day 28 for 14 consecutive days. Despite a 2-fold decrease in the administered dose of peptide inhibitor, therapeutic treatment with IA31-LPC1 resulted in the observed anti-arthritic efficacy (Figure 2B), comparable to that observed for 25 mg/kg free IA9 (Figure 2A) and the positive control (Figure 2B). The therapeutic effect of 13 mg of GA31/kg GA31-LPC1, while significant compared to vehicle-treated mice, was significantly lower compared to that of the positive control (Figure 2B). In contrast, a significant decrease in the rate of disease progression was observed in the first several days of treatment with GA31-LPC3, which later disappeared (Figure 2B). Interestingly, in our previous studies in the prophylactic CIA mouse model [30], LPC of similar size (140 nm and PDI < 0.2), prepared using POPC, cholesterol, cholesteryl oleate, and an equimolar mixture of two trifunctional TREM-1 inhibitory peptides GA31 and GE31 (GA/E31-LPC), delayed and reduced arthritis progression exerting persistent prophylactic anti-arthritic effects that was comparable to that of the positive control. In line with the findings reported for dexamethasone-loaded liposomes of different sizes [57], our data likely indicate that the larger size of GA31-LPC3, compared to that of GA31-LPC1 and IA31-LPC1, may prevent their efficient accumulation at the local (joint) inflammatory sites. Systemic and local events in inflammatory arthritis are thought to be discrete processes, driven by multiple mediators with distinct temporospatial profiles [58,59]. Thus, when given before arthritis begins, the 140 nm-sized TREM-1 inhibitory LPC formulations may effectively prevent CIA, most likely by means of inhibiting systemic inflammatory response [30]. However, when injected after onset of joint inflammation, while initially highly effective, these formulations rapidly lose their anti-arthritic efficacy (Figure 2B), probably due to their inability to access the joint and suppress local inflammation.

In terms of body weight, administration of IA9 and IA31-LPC1 resulted in an increase in body weight in contrast to the decrease observed in vehicle- or prednisolone-treated mice with CIA (Figure 2C,D), suggesting a good tolerability of TREM-2 inhibitory formulations. 

In summary, these findings collectively indicate that, in the current study, IA9 and IA31-LPC1 exerted anti-inflammatory and anti-arthritic therapeutic effects comparable to those of prednisolone used as a positive control, thereby providing further experimental in vivo evidence of the usability of the SCHOOL model to design clinically relevant ligand-independent peptide inhibitors of MIRRs [48,60]. Incorporation of IA9 sequence into macrophage-specific LPC substantially increased their therapeutic efficacy, probably because of its targeted delivery to the inflammatory sites and/or the prolonged circulatory half-life of the peptide afforded by this strategy.

### 2.2. Reduction of Joint Damage and Bone Erosion in CIA 

**Figure 3:**
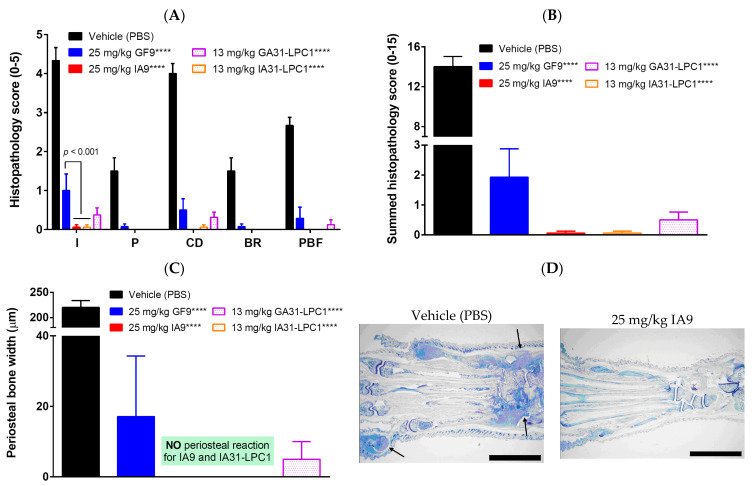
Effect of therapeutic treatment with free and LPC-bound IA9 and GF9 sequences on histopathological scores of inflammation (I), pannus (P), cartilage damage (CD), bone resorption (BR), and periosteal new bone formation (PBF) (**A**), summed histopathological score (**B**) and periosteal bone width (**C**). No periosteal reaction was observed in IA9- and IA31-LPC1-treated mice (**C**). Photomicrographs of hind paws of vehicle- and IA9-treated arthritic mice stained with toluidine blue (animals with the approximate mean summed paw score for the group were selected) (**D**). Arrows identify representative affected joints. The scale bars at the bottom right of the images indicate 5 mm. Data are shown as mean ± SEM (*n* = 8). ****, *p* < 0.0001 versus vehicle.

To gain insight into in situ pathological processes and determine whether therapeutic treatment of CIA mice with free and LPC-bound TREM-2 inhibitory IA9 sequences reduces chronic inflammation of synovial tissue, pannus formation, cartilage destruction, and bone erosion, we next examined the histopathology of six joints from each animal including fore paws, hind paws, and knees (Figure 3). 

Vehicle-treated arthritic mice had histopathology changes, consistent with those seen in type II CIA, including infiltration of synovium and periarticular tissue with neutrophils and mononuclear inflammatory cells (inflammation), marginal zone pannus and bone resorption and cartilage damage (proteoglycan loss, chondrocyte death and collagen matrix destruction). Histopathological parameters in arthritic mice treated with 2.5 mg/kg IA9 or GF9, as well as with 25 mg/kg IA9-G or GF9-G, starting day 28 for 14 consecutive days did not differ significantly from those observed in vehicle-treated mice (data not shown). In contrast, therapeutic treatment of CIA mice with higher doses of IA9 or GF9 (25 mg/kg), as well as with 13 mg/kg IA31-LPC1 or 13 mg/kg GA31-LPC1, overall significantly reduced all six-joint mean histopathological parameters compared to vehicle-treated mice (Figure 3). 

When compared to vehicle-treated mice, mice therapeutically treated with 25 mg/kg IA9 or 13 mg/kg IA31-LPC1 had significantly reduced inflammation (99 and 96% reduction, respectively), pannus formation (100% for both agents), cartilage damage (100 and 98%, respectively), bone resorption (100% for both agents), and periosteal bone formation (100% for both agents) (Figure 3A). A similar tendency was observed in mice treated with either 25 mg/kg GF9 or 13 mg/kg GA31-LPC1 (Figure 3A). Interestingly, IA9 and IA31-LPC1 were significantly more efficient than GF9 in suppressing inflammation in terms of neutrophils and mononuclear inflammatory cells infiltrated into the synovium and periarticular tissue (Figure 3A).

In line with our clinical data (Figure 2A,B), we observed a 99–100% reduction of summed histopathological scores in arthritic mice therapeutically treated with either 25 mg/kg IA9 or 13 mg/kg IA31-LPC1 compared to vehicle-treated mice (Figure 3B). While not statistically significant, there was a trend toward higher efficacy with IA9 or IA31-LPC1 than with GF9 or GA31-LPC1 at the same doses in one study. No histopathological changes were observed in arthritic mice treated with either a lower dose of IA9 (2.5 mg/kg) or control peptide (25 mg/kg IA9-G) compared to vehicle-treated mice (data not shown). 

No or little periosteal reaction was observed in arthritic mice therapeutically treated with IA9, IA31-LPC1, GF9, and GA31-LPC1 (Figure 3C). The lack of bone resorption and periosteal reaction in IA9- and IA31-LPC1-treated mice (Figure 3A,C) suggests that, along with inhibiting local inflammatory cells, these agents may directly inhibit TREM-2 expressed on osteoclasts, the cells that are of central importance in the structural damage of chronic inflammatory joint disease [61]. The hind paw joints of vehicle-treated arthritic mice showed inflammation and destruction of articular structures, whereas the joints of IA9-treated CIA mice showed retained structure with no lesions (Figure 3D). 

Thus, in line with clinical findings, the histopathology data demonstrated that therapeutic treatment with TREM-2 and TREM-1 inhibitory peptides (IA9 and GF9, respectively) as well as with targeted LPC-bound formulations of IA9 and GF9 sequences (IA31-LPC1 and GA31-LPC1, respectively) significantly reduced joint damage and bone erosion in CIA in a specific and dose-dependent manner.

### 2.3. Reduction of Plasma and Joint Proinflammatory Cytokine Levels in CIA 

In experimental and clinical arthritis, TREM-1 is well known to mediate the release of proinflammatory cytokines and chemokines, including CSF-1 [27,30,62,63,64], that are all implicated in RA disease pathogenesis [65]. In contrast, the role of TREM-2 in RA is not yet well elucidated. TREM-2 is widely thought to function as an immunomodulatory receptor, which negatively regulates inflammation [34,66,67]. Contrary to this understanding, TREM-2 is upregulated in active RA synovium and subsequently downregulated in inactive RA, suggesting a role of TREM-2 as a positive regulator of RA-induced inflammation [43]. 

**Figure 4:**
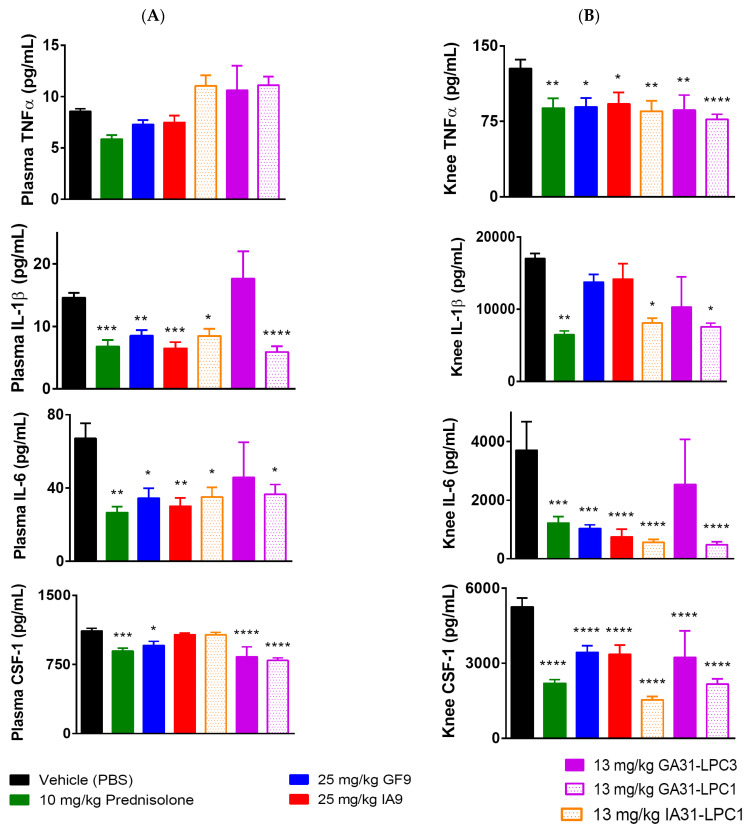
Effect of therapeutic treatment with free and LPC-bound IA9 and GF9 sequences on plasma (**A**) and joint (knee) (**B**) levels of proinflammatory cytokines. Data are shown as mean ± SEM (*n* = 12). *, *p* < 0.05; **, *p* < 0.01; ***, *p* < 0.001; and ****, *p* < 0.0001 versus vehicle.

To further elucidate the molecular mechanisms underlying the significant reduction in CIA severity observed in mice treated with IA9 and IA31-LPC1 rationally designed to inhibit TREM-2, we next analyzed the plasma and joint (knee) proinflammatory cytokine levels on day 42 at the end of the study (Figure 4). Plasma and knee tissue of mice treated with the TREM-1 inhibitory formulations used in this study (GF9, GA31-LPC1 and GA31-LPC3) were assayed comparatively. 

No differences in plasma levels of TNFα were observed on day 42 in all treatment groups, including prednisolone-treated mice, as compared to vehicle-treated controls (Figure 4A). In contrast, treatment either with oral prednisolone or i.p. administered IA9, GF9, IA31-LPC1, GA31-LPC1 or GA31-LPC3 resulted in significantly reduced knee levels of TNFα. (Figure 4B). Interestingly, plasma and knee levels of IL-1β and IL-6 in mice treated with GA31-LPC3 did not differ from those in vehicle-treated controls (Figure 4A,B). Plasma levels of IL-1β and IL-6 in mice treated with prednisolone, IA9, IA31-LPC1, GF9 and GA31-LPC1, but not in mice treated with GA31-LPC3, were significantly reduced compared to those in vehicle-treated controls (Figure 4A). In contrast, no differences in knee levels of IL-1β were observed between mice treated with vehicle, IA9 or GF9 (Figure 4B). 

Therapeutic treatment with prednisolone, GF9, GA31-LPC1 or GA31-LPC3, but not IA9 or IA31-LPC1, decreased plasma levels of CSF-1 compared to vehicle-treated mice (Figure 4A). However, marked reduction of knee levels of CSF-1 was observed in all treatment groups, with groups treated with IA31-LPC1 and GA31-LPC1 presenting the greatest decrease (Figure 4B). No significant differences were observed in plasma and knee cytokine levels in CIA mice treated with 2.5 mg IA9 or GF9 as well as with 25 mg/kg IA9-G or GF9-G, compared to those in vehicle-treated mice (data not shown).

Thus, cytokine data indicated that in the therapeutic CIA model, TREM-2 and TREM-1 inhibitory peptides (IA9 and GF9, respectively), as well as targeted LPC-bound formulations of these peptide sequences, reduced plasma and/or joint proinflammatory cytokine levels in a specific and dose-dependent manner. 

### 2.4. Immunohistochemical Analysis for F4/80, CD68, Collagen IV, TREM-1 and TREM-2

To further characterize the anti-inflammatory and anti-arthritic effects exhibited in this study by the peptide sequence IA9 and its LPC-bound formulation IA31-LPC1, we next performed immunohistochemical (IHC) staining of joint tissues for F4/80, CD68, collagen IV, TREM-1 and TREM-2. Joint tissues of CIA mice treated with GF9 and GA31-LPC1 were analyzed comparatively.

**Figure 5:**
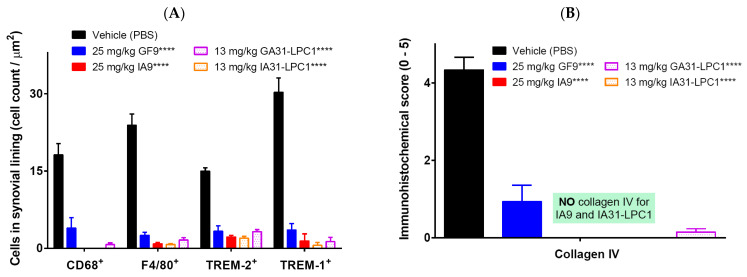
Immunohistochemical analysis of collagen-induced arthritic joints and effect of therapeutic treatment with free and LPC-bound IA9 and GF9 sequences on the number of CD68-, F4/80-, TREM-2- and TREM-1-positive cells (**A**) and collagen IV immunostaining score (**B**). Data are shown as mean ± SEM (*n* = 8). ****, *p* < 0.0001 versus vehicle.

In the synovial lining of arthritic joints of vehicle-treated mice, the mean CD68-, F4/80+, TREM-2, and TREM-1-immunopositive cell count varied from 15 to 30, depending on the marker (Figure 5A). In line with clinical and histopathological findings, all treatment groups exhibited significant reduction of CD68- and F4/80-immunopositive cell counts, compared to those of the vehicle-treated control group, with the effect being most pronounced for IA9 and IA31-LPC1 (Figure 5A). No CD68-immunopositive cells were observed in joints of mice treated either with IA9 or IA31-LPC1 (Figure 5A). TREM-1-stained sections had distinctive and fairly easy to quantify immuno-positive macrophages in, and around, the inflamed joints, and TREM-2-stained sections had distinctive, and fairly easy to quantify, immuno-positive osteoclasts and macrophages lining the endosteal and periosteal surfaces of bone. Therapeutic treatment with IA9, GF9, IA31-LPC1 or GA31-LPC1 resulted in significant reduction of TREM-1- and TREM-2-immunopositive cell counts compared to those of vehicle-treated mice (Figure 5A).

A similar tendency was observed for collagen IV with significant increase in collagen IV immunostaining in vehicle-treated mice with CIA. Therapeutic treatment with GF9 and GA31-LPC1 resulted in significant reduction of collagen IV immunostaining score, compared to that of vehicle-treated mice (Figure 5B). No collagen IV was detected in joints of mice treated with IA9 and IA31-LPC1.

Thus, IHC findings suggest that in the therapeutic CIA model, TREM-2 and TREM-1 inhibitory peptides (IA9 and GF9, respectively), as well as targeted LPC-bound formulations of these peptide sequences, significantly reduced the number of inflammatory cells in the synovial membrane of the joints, with IA9 peptide sequences being most effective. 

## 3. Discussion

Both autoimmune and systemic inflammatory responses play a role in development and progression of RA [68]. Proinflammatory cytokines are key drivers of inflammation in RA, and multiple cytokine-blocking agents, including orally active inhibitors, neutralizing antibodies, soluble receptors, or receptor antagonists, have been tested in patients with RA [69,70]. However, inadequate response and safety concerns, especially the potential for serious infections and malignancy, remain for TNFα and other cytokine blockers [20,71,72,73]. This makes a search for new therapeutic approaches to RA an area of great clinical importance. Among these approaches, targeting myeloid cells, that play a central role in the pathogenesis of RA [10,11], represents a promising perspective in this disease [74].

Despite the central role of TREM-2 in Alzheimer’s disease, metabolic syndrome, cancer, and other diverse pathologies causing it to attract considerable recent attention [36], its role in RA is not well understood. Some studies have reported upregulation of TREM-2 in active RA synovium [43] and overexpression of TREM-2 in the synovial tissue of rats with CIA [44], suggesting blockade of TREM-2 signaling or depletion of TREM-2-expressing myeloid cells from synovium as potential anti-arthritic approaches. In contrast, other studies have indicated a protective function for TREM-2-expressing synovial tissue macrophages in RA [75], which questions the hypothetical advantage of TREM-2 blocking strategy.

To our best knowledge, the present study is the first to demonstrate that TREM-2 inhibition is therapeutically effective against CIA in mice, suggesting TREM-2 as a promising target for treating RA. We showed that treatment with rationally designed ligand-independent TREM-2 inhibitory peptide sequence IA9, either in the form of free peptide or as a part of trifunctional peptide IA31 formulated into macrophage-targeted LPC (IA31-LPC1), remarkably reduced the clinical arthritic score to an extent comparable to that of prednisolone used as positive control in this study. Importantly, therapeutic treatment with IA9 or IA31-LPC1 did not lead to disease-associated weight loss in CIA mice, in contrast to that observed in vehicle- or prednisolone-treated groups. Using cytokine, histopathological and IHC analyses, we further demonstrated that therapeutic treatment with IA9 and IA31-LPC significantly reduced systemic inflammatory response and inflammatory macrophage joint infiltration, as well as joint inflammation, pannus, cartilage damage, bone resorption, and periosteal bone formation, compared to vehicle-treated controls. As predicted by the SCHOOL mechanisms of TREM-2 signaling [46,47], no therapeutic effect was observed for control peptide IA9-G with lysine replaced by glycine, suggesting specificity of this effect. Overall, our study lays the premise that ligand-independent peptide therapies targeting TREM-2 signaling may provide a novel therapeutic strategy in treating RA.

There is one major challenge that complicates our understanding of the molecular mechanisms of TREM-2 signaling and its acting as pro- [39,40,41,42] or anti-inflammatory [33,34,35,36,37,38] regulator in inflammatory diseases. Despite TREM-2 binding to a set of potential ligands that are distinct from those recognized by TREM-1, an established inflammation amplifier [32], TREM2 and TREM-1 both signal through the same signaling partner DAP-12 (Figure 1B). In addition, certain macrophages and neutrophils express both TREM-1 and TREM-2 [26], further complicating this picture. Possibly, depending on the cell type involved, TREM-1 and/or TREM-2 ligation might activate different cytoplasmic adaptors, producing different outcomes, as suggested for osteoclasts, monocyte/macrophages and dendritic cells [42]. In this study, we showed that in experimental arthritis, inhibiting TREM-2 ameliorated arthritis and significantly reduced cartilage and joint damage, similarly to what happens when inhibiting TREM-1. Further studies are needed to differentiate the precise mechanisms of the anti-inflammatory and anti-arthritic effects of TREM-2 and TREM-1, as well as to investigate whether concurrent inhibition of TREM-2 and TREM-1 signaling would be more effective than inhibition of either pathway alone.

Previously, we demonstrated that ligand-independent TREM-1 inhibitory functionality can be combined in one sequence with seemingly unrelated functionalities of other peptides resulting in multifunctional peptides GA31 and GE31 capable of self-assembling into targeted LPC upon interaction with lipids. We further demonstrated that these LPC can therapeutically inhibit TREM-1 in various inflammatory diseases, including CIA [30], pancreatic cancer [76], retinopathy of prematurity [77], and alcoholic liver disease [78]. In the present study, we extended this concept to design a multifunctional peptide IA31 with the predicted capability to inhibit TREM-2 and showed that nanosized IA31-LPC1 particles formed upon interaction of IA31 with lipid exhibited significant anti-inflammatory and anti-arthritic activities in the therapeutic CIA mouse model. This further supports the combinatorial “molecular Lego” approach to designing multifunctional peptide therapies by combining the functionality of ligand-independent peptide inhibitors of cell receptors [48] with functionalities of other peptides, such as the native, or rationally modified, amphipathic peptide sequences of apo A-I used in this study and in our previous studies [30,52,76,77,79].

Here, we showed that, as revealed by histological and proinflammatory cytokine analyses, TREM-2 inhibitory peptide sequence IA9 in the form of free peptide or formulated into targeted IA31-LPC1 formulations exhibited a high efficacy in suppressing local (joint) inflammation and that, histologically, the observed anti-inflammatory effect of IA9 and IA31-LPC1 was significantly higher than that observed for TREM-1 inhibitors GF9 and GA31-LPC1. This is in line not only with findings in RA showing that TREM-2 is highly upregulated in active but not inactive RA synovium [43], but also with studies in IBD [42,80] that reported high levels of TREM-2 in the inflamed mucosa of patients with IBD and the virtual absence of TREM-2 in colon samples of healthy donors. Further, TREM-2 is expressed by tumor-associated macrophages (TAMs) in various tumor types [81] and plays an important role in tumor immunity [82]. In experimental cancer, inhibition of TREM-2 with anti-TREM-2 blocking monoclonal antibody (mAb) not only exhibits a robust antitumor effect when used as single-agent therapy, but also significantly improves the efficacy of immune checkpoint blockade (ICB) therapy when used in combination with anti-programmed cell death protein-1 (PD-1) treatment [81,83]. Collectively, this suggests that a rationally designed ligand-independent TREM-2 inhibitory peptide sequence IA9 can be a promising alternative to ligand-dependent anti-TREM-2 mAbs to modulate local inflammation in the management of RA, IBD, cancer and, probably, other inflammatory diseases. Considering the therapeutic efficacy of both TREM-2- and TREM-1-targeting approaches in various inflammation-associated diseases, one may suggest that concurrent targeting of TREM-2 and TREM-2 would exhibit a synergistic effect in treating these diseases. Further studies are in progress to confirm this hypothesis.

In summary, we showed the effectiveness of the TREM-2-inhibiting approach in the treatment of CIA, as demonstrated by the significant decrease in clinical signs of arthritis, joint inflammation and destruction, inflammatory cell infiltration of joint tissues, and proinflammatory cytokine levels in the plasma and joints. We also demonstrated that TREM-2 inhibitory peptide sequence IA9 is therapeutically effective, not only in the form of free peptide, but also as a part of a multifunctional peptide IA31 incorporated into LPC formulations for its targeted delivery to the inflammation sites. 

## 4. Materials and Methods

### 4.1. Chemicals, Lipids and Peptides

Sodium cholate, prednisolone 21-hemisuccinate sodium salt and other chemicals were purchased from Sigma Aldrich Company (St. Louis, MO, USA). Bovine type II collagen was ordered from Chondrex, Inc. (Woodinville, WA, USA). 1-palmitoyl-2-oleoyl-sn-glycero-3-phosphocholine (POPC) and cholesterol were purchased from Avanti Polar Lipids (Alabaster, AL, USA). Cholesteryl oleate was purchased from Nu-Chek Prep, Inc. (Elysian, MN, USA). The following two synthetic 9-mer peptides were ordered from Bachem Americas, Inc. (Torrance, CA, USA): GFLSKSLVF (human TREM-1_213–221_, GF9) and GFLSGSLVF (GF9-G). Two 9-mer peptides. IFLIKILAA (human TREM-2_182–190_, IA9) and IFLIGILAA (IA9-G), and two 31-mer peptides with sulfoxidized methionine residues, GFLSKSLVFPLGEEM(O)RDRARAHVDALRTHLA (GA31) and IFLIKILAAPLGEEM(O)RDRARAHVDALRTHLA (IA31), were synthesized by Ambiopharm Inc. (Beech Island, SC, USA). All peptides were purified by reversed-phase high-performance liquid chromatography (RP-HPLC), and their purity and net peptide content were confirmed by analytical RP-HPLC, mass spectrometry and amino acid analysis. 

### 4.2. Lipopeptide Complexes

Lipopeptide complexes (LPC) of oxidized peptides GA31 and IA31 (GA31-LPC and IA31-LPC, respectively, both of spherical shape) were synthesized by substantially using the sodium cholate dialysis procedure, as previously described [30]. The initial molar ratio for complexes of oxidized GA31 with 3 lipids (GA31-LPC3) was 125:6:2:1:210, corresponding to POPC:cholesterol:cholesteryl oleate:GA31:sodium cholate. The initial molar ratio for complexes of oxidized GA31 and IA31 with 1 lipid (GA31-LPC1 and IA31-LPC1, respectively) was 125:1:210 corresponding to POPC:peptide:sodium cholate. All obtained LPC formulations of GA31 and IA31 were purified and characterized as described previously [30]. Mean LPC size was determined by dynamic light scattering (DLS) with a DynaPro-99-E-50 instrument. The polydispersity index (PDI) that can vary from 0 (monodisperse) to 1.0 (polydisperse) was used to evaluate LPC size distribution.

### 4.3. Therapeutic Collagen-Induced Arthritis (CIA) Model

All animal experiments were performed by Washington Biotechnology, Inc. (WBI; Baltimore, MD, USA). Male DBA/1J mice (7–9 weeks old) were purchased from Jackson Laboratory (Bar Harbor, MA, USA) and maintained under specific pathogen-free (SPF) conditions with food and water ad libitum. CIA was induced by immunization with bovine type II collagen as previously described [30,79]. Briefly, mice were weighed and injected subcutaneously at the base of the tail with 50 μL of Freund’s complete adjuvant containing 100 μg of bovine type II collagen (2 mg/mL final concentration) on day 0. Mice were boosted on day 21 with the same dose of bovine type II collagen, but incomplete Freund’s adjuvant was used to make the emulsion. Mice were weighed weekly and scored for signs of arthritis daily. Each paw was scored as follows: 0: no visible effects of arthritis; 1: edema and/or erythema of one digit; 2: edema and/or erythema of 2 joints; 3: edema and/or erythema of more than 2 joints; 4: severe arthritis of the entire paw and digits including limb deformation and ankylosis of the joint. The sum of all four scores was recorded as the arthritic score with the maximum possible value of 16. Starting day 28, the mice were i.p. injected daily for 14 consecutive days with GF9 (2.5 or 25 mg/kg), GF9-G (25 mg/kg), IA9 (2.5 or 25 mg/kg) or IA9-G (25 mg/kg) as well as with IA31-LPC1, GA31-LPC3 or GA31-LPC1 (all at a dose of 13 mg of peptide/kg) or with vehicle (phosphate-buffered saline, pH 7.4; PBS). The positive treatment control for these experiments was oral prednisolone administered at a dose of 10 mg/kg daily for 14 consecutive days starting day 28. Once the dosing regimen was initiated, the mice were weighed and scored for signs of disease three times a week and prior necropsy (day 42). On day 42, mice were euthanized for necropsy. 

### 4.4. Histological Assessment

At the end of study, fore paws, hind paws, and knees were harvested, fixed in 10% neutral buffered formalin (NBF) for 1–2 days, and then decalcified in 5% formic acid for 4–5 days before standard processing for paraffin embedding. Sections (8 μm) were cut and stained with toluidine blue (T blue) essentially as described [84]. Hind paws, fore paws, and knees were embedded and sectioned in the frontal plane. Six joints from each animal were processed for histopathological evaluation. The joints were then evaluated by a board-certified veterinary pathologist using light microscopy using 0–5 scale for inflammation, pannus formation, cartilage damage, bone resorption, and periosteal new bone formation as previously reported [30]. A summed histopathology score (sum of five parameters, 0–25 scale) was also determined. 

### 4.5. Plasma and Joint Cytokine Analysis 

Plasma and joint tissues were collected by the end of day 42. The knees were minced and flash frozen in liquid nitrogen and stored at −80 °C. Then, the collected knees were thawed to 4–8 °C and homogenized in 1 mL 2X cell lysis buffer (Cell Signaling Technology, Inc.; Danvers, MA, USA) followed by sonication. The homogenates were centrifuged for 15 min and the supernatants were stored at −80 °C until analyzed. Cytokines were analyzed in the collected plasma and knee homogenates using ELISA kits (R&D Systems, Minneapolis, MN, USA) according to the manufacturer’s instructions.

### 4.6. Immunohistochemical Analysis

Immunohistochemical (IHC) staining in formalin-fixed, paraffin-embedded (FFPE) mouse limb sections was conducted using the Bond RXm platform (Leica Biosystems, Deer Park, IL, USA) and antibodies to F4/80 (Absolute Antibody, [CI:A3-1], Ab00106-23.0), CD68 (Abcam, ab125212), collagen IV (Abcam, ab6586), TREM-1 (LS-Bio, LS-C312743) and TREM-2 (LS-Bio, LS-C489619). Antibody binding was detected using an horseradish peroxidase (HRP)-conjugated secondary polymer, followed by chromogenic visualization with diaminobenzidine (DAB). A hematoxylin counterstain was used to visualize nuclei. IHC staining of collagen IV was scored on a scale of 0 to 5 based on increases in the percent area of disease-associated staining: 0: normal; 0.5: very minimal, <1% of area at risk affected; 1: minimal, approximately 1–10% of area at risk affected; 2: mild, approximately 11–25% of area at risk affected; 3: moderate, approximately 26–50% of area at risk affected; 4: marked, approximately 51–75% of area at risk affected; and 5: severe, approximately 76–100% of area at risk affected. Areas of normal background staining (distal digit nail beds, skin, periosteum, endosteum, and osteocyte lacunae) were not included in the % area affected/scores. CD68, F4/80, TREM-1, and TREM-2 immuno-positive cells were counted at 400x magnification in a defined square micron area in synovium/exudate (10 × 100 units = 25 × 250 μm = 6250 μm^2^), and the percentage of positive cells was calculated.

### 4.7. Statistical Analysis

All statistical analyses were performed using GraphPad Prism 6.0 software (GraphPad, La Jolla, CA, USA). Results are expressed as the mean ± SEM. Statistical differences were analyzed using analysis of variance with Bonferroni adjustment. *p* values less than 0.05 were considered significant. 

### 4.8. Sequence Accession Numbers 

Accession numbers (UniProtKB/Swiss-Prot knowledgebase, http://www.uniprot.org/, accessed on 22 July 2022) for the protein sequences discussed in this article are as the follows: human TREM-2, Q9NZC2; human TREM-1, Q9NP99.

## 5. Conclusions

In this study, we demonstrated that TREM-2 can be a promising target in therapy of RA. We further provided compelling in vivo evidence of the therapeutic potential of targeting TREM-2 in RA using the TREM-2 inhibitory peptide sequence IA9 rationally designed using the SCHOOL model of cell signaling. This further expands the applicability of our SCHOOL concept of ligand-independent inhibition of various cell receptors in multiple diseases and disorders, where blockade of these receptors is of clinical value.

## Data Availability

The data obtained in this study are available in the article.

## Abbreviations

RA	Rheumatoid arthritis
IL	Interleukin
TNF	Tumor necrosis factor
M-CSF	Macrophage-colony stimulating factor
CIA	Collagen-induced arthritis
SEM	Standard error of the mean
TREM-1	Triggering receptors expressed by myeloid cells 1
TREM-2	Triggering receptors expressed by myeloid cells 2
DAP-12	DNAX-activating protein of 12 kDa
SCHOOL	Signaling chain homo-oligomerization
MIRR	Multichain immune recognition receptor
LPC	Lipopeptide complex
HDL	High density lipoproteins
Apo	Apolipoprotein
SR-A	Type A scavenger receptor
IP	Intraperitoneally
I	Inflammation
P	Pannus
CD	Cartilage damage
PBF	Periosteal bone formation
POPC	1-Palmitoyl-2-oleoyl-sn-glycero-3-phosphocholine
IBD	Inflammatory bowel disease
mAb	Monoclonal antibody
ICB	Immune checkpoint blockade
PD-1	Programmed cell death protein 1
RP-HPLC	Reversed-phase high-performance liquid chromatography
DLS	Dynamic light scattering
PDI	Polydispersity index
SPF	Specific pathogen-free
PBS	Phosphate-buffered saline
NBF	Neutral buffered formalin
T blue	Toluidine blue
DAB	Diaminobenzidine
IHC	Immunohistochemistry
HRP	Horseradish peroxidase
ELISA	Enzyme-linked immunosorbent assay
FFPE	Formalin-fixed, paraffin-embedded
